# Male Breast Cancer: Clinical, Demographical, and Pathological Features in a Cohort of 41 Patients

**DOI:** 10.7759/cureus.17812

**Published:** 2021-09-08

**Authors:** Tolga Koseci, Veysel Haksöyler, Polat Olgun, Mahmut Bakır Koyuncu, Berna Bozkurt Duman, Timuçin Çil

**Affiliations:** 1 Medical Oncology, Adana City Training and Research Hospital, Adana, TUR; 2 Medical Oncology, Adana Medline Hospital, Adana, TUR; 3 Department of Medical Oncology, Near East University, Lefkoşa, CYP; 4 Hematology, Adana City Training and Research Hospital, Adana, TUR; 5 Oncology, University of Health Sciences, Adana City Training and Research Hospital, Adana, TUR; 6 Medical Oncology, University of Health Sciences, Adana City Training and Research Hospital, Adana, TUR

**Keywords:** overall survival, notthingam prognostic index, male, breast cancer, progression - free survival

## Abstract

Background and objective

Male breast cancer (MBC) is a rare malignancy, and it accounts for less than 1% of all cancers in men. The pathogenesis of MBC remains unclear, with most available data obtained from single-center studies and retrospective series. The aim of this study was to share our experiences of MBC cases and to describe the characteristics of MBC patients.

Materials and methods

We retrospectively reviewed the records of 41 MBC cases and recorded the pathological, clinical, and demographic features of the patients. Data on progression-free survival (PFS) and overall survival (OS) were also recorded.

Results

The mean age of the patients was 64.1 ± 10.0 years. The most common histopathological subtype was invasive ductal carcinoma. Hormone receptor positivity was detected in 39 (95.1%) patients. Human epidermal growth factor receptor 2 (HER2) positivity was present in five (12.2%) patients. Most of the patients had early-stage disease. Surgery was the treatment of choice for most primary tumors. Thirty-nine (95.1%) patients received hormonotherapy, and 21 (51.2%) received systemic chemotherapy. OS was found to be 126.4 months and PFS was 83.2 months. The OS and PFS time in patients with a Nottingham Prognostic Index (NPI) score of <5.4 were longer than those with an NPI score of >5.4.

Conclusion

The hormone receptor status of most of the MBC patients was positive, and their HER2 status was negative. A multimodality approach was associated with longer survival, which has been reported in female patients with breast cancer as well. The NPI score is a useful tool for predicting survival time in MBC patients.

## Introduction

Male breast cancer (MBC) is a rare condition and constitutes less than 1% of all breast cancers. Various factors, such as radiation exposure, cirrhosis, Klinefelter syndrome, genetics, and obesity, play a role in the etiology of MBC [1]. The incidence of MBC increases with age. The BRCA-2 mutation is more common than the BRCA-1 mutation in cases of genetic inheritance of MBC [2]. The lifetime risk of breast cancer is 1-5% in MBC patients with the BRCA-1 mutation and 5-10% in MBC patients with the BRCA-2 mutation [3].

Tumor diameter and axillary lymph node involvement are the prognostic factors in male and female breast cancer patients. Differences between MBC and female breast cancer include the ratio of hormone positivity, with a higher ratio observed in MBC patients compared to female breast cancer patients. In contrast, the rate of human epidermal growth factor receptor 2 (HER2) positivity in MBC patients is lower than that in female breast cancer patients [4,5]. A radical mastectomy is usually the treatment of choice. The indications for radiotherapy and chemotherapy in MBC patients are similar to those in female breast cancer patients.

The Nottingham Prognostic Index (NPI) was first described in 1987 and is calculated using the tumor size, tumor grade, and lymph node status [6]. It is a histopathological grading model, which characterizes tumor behavior better than the TNM staging system. NPI is not used for metastatic disease.

Given the rarity of MBC, the aim of the present study was to share our experiences of MBC cases and to present clinical, pathological, and demographic data on patients with a diagnosis of MBC who were followed up at our clinic.

## Materials and methods

Study sample

MBC patients who were followed up at the Adana City Training and Research Hospital Cancer Center between January 2010 and May 2020 were included in this study. In total, 41 patients were included in this retrospective cohort analysis. The relapse time, metastasis sites, treatments, pathological features, and demographic features of the patients were recorded. Overall survival (OS) and progression-free survival (PFS) were also recorded.

All the patients with metastatic disease underwent radiological studies. Bone scintigraphy was performed in patients with symptoms or laboratory findings. The staging of the patients was done according to the TNM system.

The pathological features recorded included the Ki-67 ratio, HER2 status, hormone receptor status, metastatic axillary lymph node involvement, and tumor grade and diameter. Immunohistochemical studies or in situ hybridization methods were used to determine HER2 expression. Patients who had >1% estrogen receptor status were considred hormone receptor-positive. The Ki-67 cut-off value was determined based on the St. Gallen guidelines, published in 2013 [7]. As per these guidelines, a cut-off value of 14% was applied for distinguishing between luminal B and luminal A breast cancer, with a Ki-67 value of <14% considered luminal A breast cancer and a Ki-67 value of ≥14% categorized as luminal B cancer.

The NPI was calculated using the following equation: NPI = 0.2 × tumor size (cm) + grade (1-3) + lymph node status (1-3) [8]. The patients were classified into three groups according to their NPI scores: ≤3.4 (good), >3.4 but ≤5.4 (moderate), and >5.4 (poor). The NPI was not calculated for patients with metastatic disease.

Statistical analysis

The SPSS Statistics version 22.0 (IBM, Armonk, NY) was used for statistical analysis. The Kolmogorov-Smirnov normality test was used to determine whether the data were normally distributed (p<0.05). Continuous variables were summarized using the mean and standard deviation, and categorical OS curves were plotted using the Kaplan-Meier method. We used a log-rank test to test the association between clinical characteristics and OS. OS was defined as death occurring after the diagnosis. PFS was defined as the time from the diagnosis to either first disease progression or death. If the patient was still alive at the last clinical evaluation, the data were censored.

Ethical approval

The study was approved by the Ethics Committee of the Adana City Training and Research Hospital. All the patients were informed about the study.

## Results

The median age of the MBC patients (n=41) in this study was 63.0 ± 10.0 years. None of the patients had a family history of MBC. A palpable mass was detected in 16 patients (39%) at the time of presentation. Metastasis was detected in 16 (39%) of the 41 patients, three of whom had metastases at the time of their diagnosis. Metastasis was diagnosed in the other 13 (31.7%) patients after the diagnosis. The sites of metastases were as follows: brain (n=1), lung and bone (n=5), lung (n=6), and bone (n=9).

An invasive ductal carcinoma was detected in 40 (97.5%) patients, and mixed histopathology was seen in one (2.5%) patient. In all the patients, MBC was diagnosed based on a true-cut biopsy. The hormone receptor status was positive in 39 (95.1%) of the 41 patients in the histopathological examination. Five (12.1%) patients were HER2-positive, and two (4.8%) patients were triple-negative. The tumor grade was grade 3 in 16 (39%) patients, grade 2 in 24 (58.5%) patients, and grade 1 in one (2.4%) patient. Tumor localization was in the right breast in 14 (34.1%) patients and in the left breast in 27 (65.8%) patients.

Surgery was the treatment of choice for the primary tumor in 39 (95.1%) cases. Thirty-seven (90.2%) of the 41 patients underwent a modified radical mastectomy, and two (4.8%) of the 41 patients underwent a simple mastectomy. An excisional biopsy was performed in only two (4.8%) patients. The mean tumor diameter was 2.89 ± 1.27 cm. The TNM stage was T3 in four (9.7%) patients, T2 in 29 (70.7%) patients, and T1 in eight (19.5%) patients. Axillary lymph node dissection was performed in 39 (95.1%) patients. Lymph node metastasis was absent in 24 (58.5%) patients. Lymph node metastasis was classified as N3 in three (7.3%) patients, N2 in two (4.8%) patients, and N1 in 11 (26.8%) patients. The lymph node status of one patient was unknown. In terms of the disease stage at the time of diagnosis, there were three (7.3%) patients with stage IV disease, six (14.6%) patients with stage III disease, 26 (63.4%) patients with stage II disease, and six (14.6%) patients with stage I disease.

None of the patients received neoadjuvant treatment. Chemotherapy was administered to 23 (56%) patients, and hormonotherapy was administered to 39 (95.1%) patients post-surgery. Twenty-one of the 39 patients who received hormonotherapy underwent systemic chemotherapy treatment. Twenty-one (51.2%) patients received radiotherapy treatment. The demographic and clinical data of the patients are shown in Table 1.

**Table 1:** Patient characteristics SD: standard deviation; OS: overall survival; PFS: progression-free survival; ALND: axillary lymph node dissection

Variables	Values (n=41)
Median age in years, ± SD (range)	63.0 ± 10.0 (45-85)
Tumor diameter (cm), mean ± SD	2.89 ± 1,27
Tumor stage, n (%)	
T1	8 (19.5)
T2	29 (70.7)
T3	4 (9.8)
Lymph node status, n (%)	
N0	24 (58.5)
N1	11 (26.8)
N2	2 (4.9)
N3	3 (7.3)
Nx	1 (2.4)
Grade, n (%)	
I	1 (2.4)
II	24 (58.5)
III	16 (39.0)
Histology, n (%)	
Ductal	40 (97.6)
Lobular	0 (0.0)
Ductal + lobular	1 (2.4)
Tumor localization, n (%)	
Right	14 (34.1)
Left	27 (65.9)
Estrogen receptor, n (%)	
Positive	39 (95.1)
Negative	2 (4.9)
Human epidermal receptor-2 status, n (%)	
Positive	5 (12.2)
Negative	36 (87.8)
Treatment, n (%)	
Chemotherapy	2 (4.8)
Hormonotherapy	39 (95.1)
Chemotherapy + hormonotherapy	21 (51.2)
Radiotherapy	21 (51.2)
Surgery, n (%)	
Mastectomy + ALND	37 (90.2)
Breast-conserving surgery + ALND	2 (4.9)
Mass excision	2 (4.9)
Metastasis side, n (%)	
Bone	9 (21.9)
Lung	6 (14.6)
Brain	1 (2.4)
Bone + lung	5 (12.1)
No metastasis	25 (60.9)
Stage at diagnosis, n (%)	
Ia	6 (14.6)
Ib	0 (0)
IIa	14 (34.1)
IIb	12 (29.1)
IIIa	2 (4.8)
IIIb	1 (2.4)
IIIc	3 (7.3)
IV	3 (7.3)
Death, n (%)	6 (14.6)
Progression, n (%)	13 (31.7)
OS, mean ± SD	126.4 ± 9.3
PFS, mean ± SD	83.2 ± 6.9

**Figure 1:**
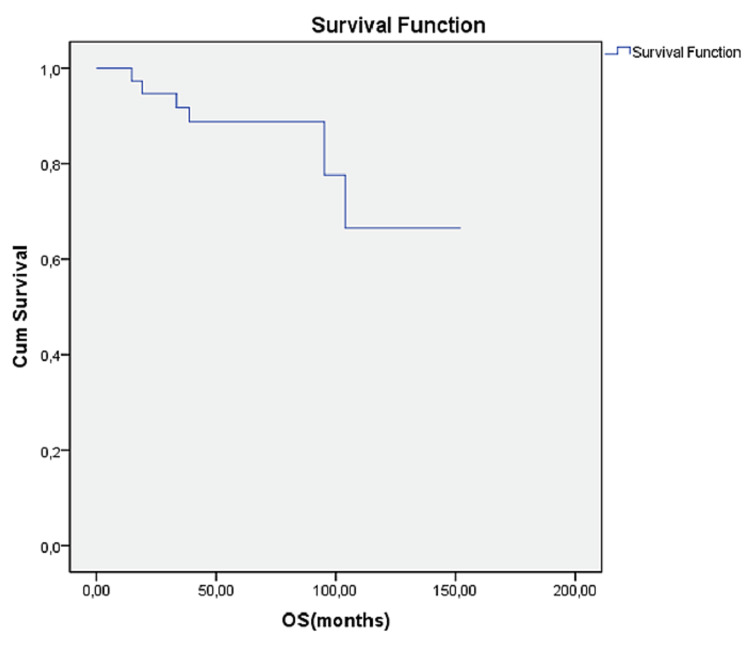
Overall survival of patients

**Figure 2:**
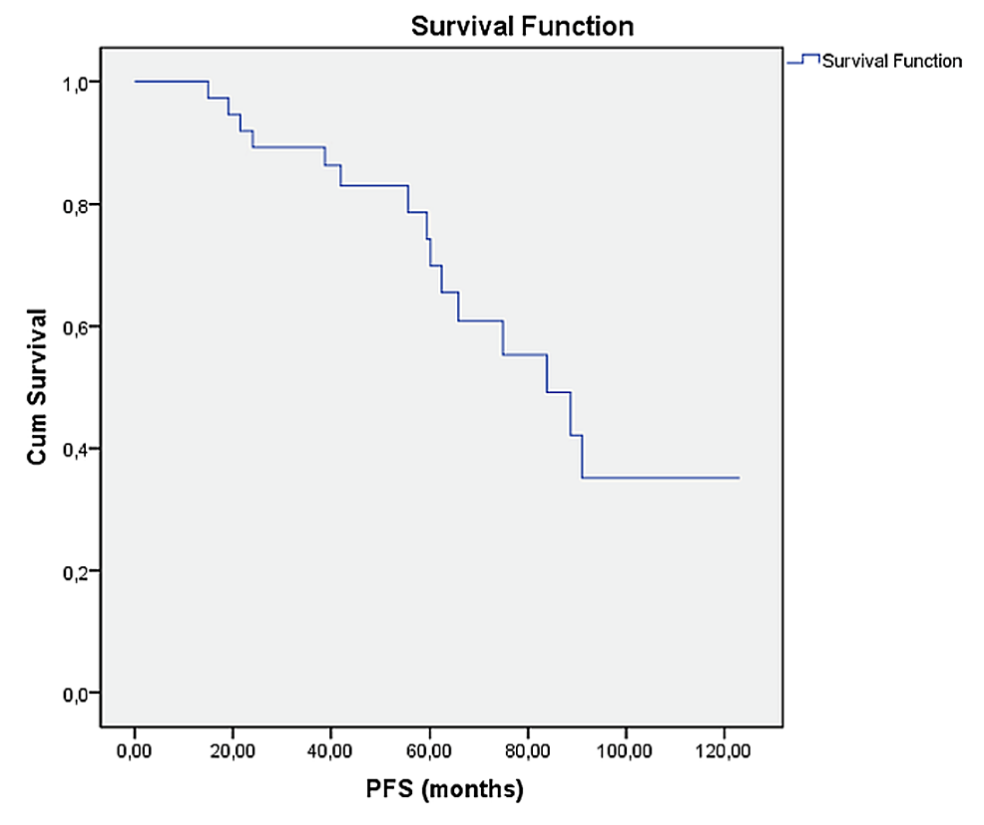
Progression-free survival of patients

At the follow-up, six (14.6%) patients were found to have succumbed to the disease, and progression was detected in 18 (43.9%) patients. The OS was 126.4 months (Figure 1) (95% confidence interval (CI): 108.12-144.7), and PFS was 83.2 months (95% CI: 69.6-96.8) (Figures 1, 2).

The patients were divided into two groups according to age (<60 years and >60 years). The tumor diameter was 2.64 cm in patients aged younger than 60 years and 3.0 cm in patients older than 60 years. The incidence of early-stage disease was similar in the two age groups. The OS of those older than 60 years was 126.1 months, whereas it was 109.2 months in those aged younger than 60 years (p>0.05). PFS was 93.7 months in patients younger than 60 years and 73.8 months in those older than 60 years (p>0.05). The histopathological characteristics and survival analysis according to age at the time of diagnosis are summarized in Table 2.

OS and PFS were longer in patients without lymph node metastasis than those with lymph node metastasis but the difference was not statistically significant (p=0.25 for OS, p=0.46 for PFS respectively). Patients with grade I-II tumors had longer OS and PFS than those with grade III tumors (p=0.56 for OS, p=0.141 for PFS respectively). Patients who had left-side tumors had longer OS and PFS compared to those with right-sided tumors (p=0.73 for OS, p=0.302 for PFS). OS and PFS results according to clinicopathological features are summarized in Table 3.

**Table 2:** Histopathologic characteristics and survival analysis related to age at diagnosis SD: standard deviation; OS: overall survival; PFS: progression-free survival

Variables	Patients aged <60 years (n=20)	Patients aged >60 years (n=21)	P-value
Tumor diameter (cm), mean ± SD	3 ± 0.95	3.1 ± 1.4	0.59
Stage at diagnosis, n			
I-II	16	16	0.92
III-IV	4	16	
ER/PR, n			0.48
Positive	18	21	
Negative	2	-	
Ki-67, n			0.86
<14	8	11	
≥14	12	10	
Luminal subtype, n			0.86
A	8	11	
B	10	10	
OS, mean ± SD	109.2 ± 8.6	126.1 ± 11.8	0.86
PFS, mean ± SD	93.7 ± 11.7	73.8 ± 6.5	0.278

**Table 3:** Overall and progression-free survival time according to clinicopathological parameters SD: standard deviation; OS: overall survival; PFS: progression-free survival; NPI: Nottingham Prognostic Index

Parameters	Total (n)	Total (%)	OS		PFS	
			Mean ± SD	P-value	Mean ± SD	P-value
Age (years)						
<60	13	31.7	109.2 ± 8.6	0.863	93.7 ± 11.7	0.278
≥60	28	68.3	126.1 ± 11.8		73.8 ± 6.5	
Lymph node metastasis						
Absent	24	60	133.7 ± 9.7	0.259	85.8 ± 7.7	0.46
Present	16	40	89.3 ± 8.6		68.8 ± 10.5	
Grade						
I-II	25	61.0	125.2 ± 9.7	0.568	91.2 ± 8.6	0.141
III	16	39.0	118.7 ± 16.2		68.7 ± 8.2	
Tumor localization						
Right	14	34.1	122.7 ± 13.2	0.736	73.1 ± 7.6	0.302
Left	27	65.9	129.0 ± 8.7		91.1 ± 9.6	
NPI						
Good-moderate	34	89.4	127.6 ± 9.6	0.200	85.8 ± 6.9	0.098
Poor	4	10.6	55.1 ± 8.8		37.1 ± 11.7	
Luminal subtype						
A	19	48.8	122.4 ± 11.0		80.7 ± 7.7	0.43
B	20	51.2	112.6 ± 19.0		70.5 ± 6.7	

## Discussion

The incidence of MBC increases with age. According to previous research, the mean age at which MBC occurs is 67-72 years. In our study, when we stratified the patients according to age (<60 years and >60 years), most of the patients were older than 60 years. The median age at the time of diagnosis in our study was 63 years. The average age of the patients in our study was consistent with that reported in the literature [9]. In previous research, males were, on average, five years older than females at the time of the diagnosis of breast cancer [10]. Differences in the average age at the time of diagnosis may be due to the age structure of the study population.

Although the NPI, luminal subtype grade, and hormone receptor positivity ratio were higher in the patients older than 60 years, the difference was not statistically significant. In addition, the tumor diameter in the MBC patients older than 60 years was larger than that in the patients younger than 60 years, but the finding was not statistically significant. Although previous research has reported more aggressive diseases in young women diagnosed with breast cancer, similar findings have not been reported in MBC patients. In our study, the OS was similar in the two age groups. Although PFS was longer in the MBC patients younger than 60 years, the difference was not statistically significant.

Invasive ductal carcinoma is the most common histopathological subtype reported in MBC patients [11]. The findings of the present study in this regard are in accordance with the literature. Invasive lobular carcinomas are rarely found in MBC patients. In the present study, mixed histopathology was detected in one patient.

The left breast is the most common site in MBC. Similar to the literature, in our study, the left breast was the most common site of the primary tumor. Lymph node metastasis is generally present at the time of diagnosis in MBC patients, and such patients usually progress to advanced-stage disease. In previous studies, 95% of patients diagnosed with MBC were diagnosed in the early stage of the disease [11,12]. Lymph node metastasis was absent at the time of diagnosis in 58% of our patients, with 78% having the early-stage disease. Although six patients had stage III disease, only three patients were metastatic.

In our study, hormone receptor positivity was detected in 95.1% of the patients, which is consistent with the literature. In previous research, HER2 positivity was detected in approximately 20% of female breast cancer patients. This ratio may vary between 3 and 40% in MBC patients [13]. This ratio was 12% in our study. Triple-negative breast cancer is rare in MBC patients [11]. In our study, two patients had triple-negative disease.

MBC is commonly sporadic. Two tumor suppressor genes, BRCA-1 and BRCA-2, play a role in DNA repair. The most important risk factor for MBC is BRCA gene mutations, with the risk of MBC higher among individuals with the BRCA-2 mutation compared to those with BRCA-1 mutation. According to previous research, the risk of breast cancer increased two-fold in males with breast cancer in first-degree relatives [14]. The family history ratio was 5-10%, and this risk increased in patients with the BRCA-2 mutation [15]. None of the patients in our study had a family history of MBC. In our study, no data were available on the BRCA-1 and BRCA-2 mutation status of the patients.

In the present study, most of the patients had tumor grades 2-3, which was consistent with the literature [13,16,17]. The tumor grade was not associated with OS or DFS. These results are in agreement with the previous research.

The NPI score is calculated using various parameters, such as the tumor diameter, tumor grade, and lymph node involvement. In our study, we used a score of 5.4 as the cut-off value for OS and PFS, in accordance with that applied in a previous study [18]. In the subgroup analysis, the OS and PFS times of the patients with an NPI score of <5.4 were longer than those of the patients with an NPI score of >5.4. However, the finding was not statistically significant.

In a previous study on 1,500 MBC patients, the median OS of patients with luminal B disease was 8.8 years versus 9.5 years for patients with luminal A disease [11]. In our study, the median OS was 10.5 years. The median OS for patients with the luminal A subtype was 10.1 years, whereas that of the patients with the luminal B subtype was 9.3 years. Differences in the numbers of patients included in the studies may explain the dissimilarity in the results.

According to previous research, the most common surgical approach in patients diagnosed with MBC was a modified radical mastectomy [19]. In our study, a modified radical mastectomy was performed in 37 of the patients. Axillary dissection was performed in 39 patients. The use of both surgical methods for primary tumors is compatible with reports in the literature.

The optimum adjuvant treatment for MBC, a rare entity, remains unclear. At present, the same drugs administered as treatments for breast cancer in females are used to treat MBC. Indications applied in the treatment of breast cancer in females are considered in treatment planning for MBC as well [3]. The drug most commonly used is tamoxifen in endocrine treatment. In our study, 39 of the patients received tamoxifen treatment. The treatment protocols used in the treatment of breast cancer in women have been applied to patients who received systemic chemotherapy and the chemotherapy drugs taxanes, doxorubicin, and cyclophosphamide. Twenty-one patients received hormonotherapy, together with systemic chemotherapy.

## Conclusions

MBC is an extremely rare entity. The treatments used for MBC are similar to those used for female breast cancer. As compared with female breast cancer patients, patients with MBC tend to be older at the time of diagnosis. The incidence of hormone receptor positivity among MBC patients is also higher than that among female breast cancer patients. The NPI score may serve as a useful indicator of OS and PFS among MBC patients.

## References

[1] Brinton LA, Carreon JD, Gierach GL, McGlynn KA, Gridley G (2010). Etiologic factors for male breast cancer in the U.S. Veterans Affairs medical care system database. Breast Cancer Res Treat.

[2] Ding YC, Steele L, Kuan CJ, Greilac S, Neuhausen SL (2011). Mutations in BRCA2 and PALB2 in male breast cancer cases from the United States. Breast Cancer Res Treat.

[3] Silvestri V, Barrowdale D, Mulligan AM (2016). Male breast cancer in BRCA1 and BRCA2 mutation carriers: pathology data from the Consortium of Investigators of Modifiers of BRCA1/2. Breast Cancer Res.

[4] Shaaban AM, Ball GR, Brannan RA (2012). A comparative biomarker study of 514 matched cases of male and female breast cancer reveals gender-specific biological differences. Breast Cancer Res Treat.

[5] Cardoso F, Costa A, Norton L (2014). ESO-ESMO 2nd international consensus guidelines for advanced breast cancer (ABC2). Breast.

[6] Todd JH, Dowle C, Williams MR (1987). Confirmation of a prognostic index in primary breast cancer. Br J Cancer.

[7] Goldhirsch A, Winer EP, Coates AS, Gelber RD, Piccart-Gebhart M, Thürlimann B, Senn HJ (2013). Personalizing the treatment of women with early breast cancer: highlights of the St Gallen International Expert Consensus on the Primary Therapy of Early Breast Cancer 2013. Ann Oncol.

[8] Fong Y, Evans J, Brook D, Kenkre J, Jarvis P, Gower-Thomas K (2015). The Nottingham Prognostic Index: five- and ten-year data for all-cause survival within a screened population. Ann R Coll Surg Engl.

[9] Gargiulo P, Pensabene M, Milano M (2016). Long-term survival and BRCA status in male breast cancer: a retrospective single-center analysis. BMC Cancer.

[10] Giordano SH, Cohen DS, Buzdar AU, Perkins G, Hortobagyi GN (2004). Breast carcinoma in men: a population-based study. Cancer.

[11] Cardoso F, Bartlett JM, Slaets L (2018). Characterization of male breast cancer: results of the EORTC 10085/TBCRC/BIG/NABCG International Male Breast Cancer Program. Ann Oncol.

[12] Anderson WF, Jatoi I, Tse J, Rosenberg PS (2010). Male breast cancer: a population-based comparison with female breast cancer. J Clin Oncol.

[13] Kornegoor R, Verschuur-Maes AH, Buerger H (2012). Molecular subtyping of male breast cancer by immunohistochemistry. Mod Pathol.

[14] Brinton LA, Richesson DA, Gierach GL, Lacey JV Jr, Park Y, Hollenbeck AR, Schatzkin A (2008). Prospective evaluation of risk factors for male breast cancer. J Natl Cancer Inst.

[15] Liede A, Karlan BY, Narod SA (2004). Cancer risks for male carriers of germline mutations in BRCA1 or BRCA2: a review of the literature. J Clin Oncol.

[16] Lacle MM, Kornegoor R, Moelans CB (2013). Analysis of copy number changes on chromosome 16q in male breast cancer by multiplex ligation-dependent probe amplification. Mod Pathol.

[17] Johansson I, Nilsson C, Berglund P (2012). Gene expression profiling of primary male breast cancers reveals two unique subgroups and identifies N-acetyltransferase-1 (NAT1) as a novel prognostic biomarker. Breast Cancer Res.

[18] Al jarroudi O, Zaimi A, Brahmi SA, Afqir S (2019). Nottingham Prognostic Index is an applicable prognostic tool in non-metastatic triple-negative breast cancer. Asian Pac J Cancer Prev.

[19] Zhou FF, Xia LP, Guo GF, Wang X, Yuan ZY, Zhang B, Wang F (2010). Changes in therapeutic strategies in Chinese male patients with breast cancer: 40 years of experience in a single institute. Breast.

